# A comparison of lesion mapping analyses based on CT versus MR imaging in stroke

**DOI:** 10.1016/j.neuropsychologia.2023.108564

**Published:** 2023-06-06

**Authors:** Margaret J. Moore, Mark Jenkinson, Ludovica Griffanti, Hanne Huygelier, Celine R. Gillebert, Nele Demeyere

**Affiliations:** a Queensland Brain Institute, University of Queensland, Brisbane, Australia; b Department of Experimental Psychology, University of Oxford, Oxford, United Kingdom; c Wellcome Centre for Integrative Neuroimaging, Oxford Centre for Functional MRI of the Brain, Nuffield Department of Clinical Neurosciences, University of Oxford, Oxford, United Kingdom; d Australian Institute for Machine Learning, University of Adelaide, Adelaide, Australia; e Wellcome Centre for Integrative Neuroimaging, Oxford Centre for Human Brain Activity, Department of Psychiatry, University of Oxford, Oxford, United Kingdom; f Brain and Cognition Unit, KU Leuven, Leuven, Belgium

**Keywords:** Lesion symptom mapping, Clinical neuroimaging, Routine imaging, Stroke

## Abstract

It is commonly asserted that MRI-derived lesion masks outperform CT-derived lesion masks in lesion-mapping analysis. However, no quantitative analysis has been conducted to support or refute this claim. This study reports an objective comparison of lesion-mapping analyses based on CT- and MRI-derived lesion masks to clarify how input imaging type may ultimately impact analysis results.

Routine CT and MRI data were collected from 85 acute stroke survivors. These data were employed to create binarized lesion masks and conduct lesion-mapping analyses on simulated behavioral data. Following standard lesion-mapping analysis methodology, each voxel or region of interest (ROI) were considered as the underlying “target” within CT and MRI data independently. The resulting thresholded z-maps were compared between matched CT- and MRI-based analyses. Paired MRI- and CT-derived lesion masks were found to exhibit significant variance in location, overlap, and size. In ROI-level simulations, both CT and MRI-derived analyses yielded low Dice similarity coefficients, but CT analyses yielded a significantly higher proportion of results which overlapped with target ROIs. In single-voxel simulations, MRI-based lesion mapping was able to include more voxels than CT-based analyses, but CT-based analysis results were closer to the underlying target voxel. Simulated lesion-symptom mapping results yielded by paired CT and MRI lesion-symptom mapping analyses demonstrated moderate agreement in terms of Dice coefficient when systematic differences in cluster size and lesion overlay are considered.

Overall, these results suggest that CT and MR-derived lesion-symptom mapping results do not reliably differ in accuracy. This finding is critically important as it suggests that future studies can employ CT-derived lesion masks if these scans are available within the appropriate time-window.

## 1 Introduction

Lesion-symptom mapping (LSM) is a powerful, and frequently employed method for establishing brain-behavior relationships (Bates et al., 2003; de Haan and Karnath, 2018). Despite this popularity, little is known about how methodological choices made when designing LSM studies may ultimately bias results. Specifically, it is unclear to what extent the imaging modality employed might impact each investigation's ability to localize neural correlates. It is critically important to establish how imaging type may impact analysis results, as this information will help guide future studies towards producing reliable and generalizable results.

Traditional LSM is a mass univariate statistical approach in which patients are grouped according to whether they have damage at a specific voxel, then behavioral scores are compared across groups (Bates et al., 2003; de Haan and Karnath, 2018; Moore, 2022). This analysis is repeated for each voxel meeting inclusion criteria, yielding a 3D statistical map highlighting voxels at which damage is significantly associated with the behavioral score. However, before conducting a LSM investigation, researchers must create lesion masks denoting the brain voxels that are damaged (Bates et al., 2003). These lesion masks are usually made by manually tracing the boundaries of stroke damage onto native space CT or MR brain scans. In general, MR scans are considered to be the preferred modality for quantifying lesion boundaries, as these scans are comparatively high-quality and clearly visualize boundaries between damaged and intact tissue (Bryan et al., 1991; Kertesz et al., 1987; Mullins et al., 2002). However, MR is not without limitations. In most countries, MR scans are not routinely conducted during acute hospitalization, meaning that many studies using MR data must use scans collected at a later (often chronic) timepoint (Mair and Wardlaw, 2014; Vert et al., 2017). This delay allows for structural reorganization, introducing potentially confounding variation into LSM analyses (de Haan and Karnath, 2018).

Additionally, a substantial portion of the stroke population exhibit contraindications to MR (Singer et al., 2004). Singer et al. (2004) found that 19.9% of acute stroke patients are ineligible for MR due to contraindications (such as metallic implants), diminished consciousness, vomiting, agitation, and hemodynamic compromise. This reduces results generalizability as analyses include a limited, non-random sample. Adding MR scanning to research protocols can further limit the number and representativeness of patients willing to participate, as some may opt to avoid the expected discomfort associated with MR. Finally, MR scanning is an expensive and time-consuming process which often strictly limits study sample size. This issue is critically important, as MR analyses with low lesion overlap have poor statistical power and are therefore highly likely to yield uninformative, false negative results (Cohen, 1992; de Haan and Karnath, 2018).

Alternatively, CT can be used for LSM. While CT has comparably worse resolution and worse tissue contrast than MR, these scans are generally more widely available (Mair and Wardlaw, 2014). CT can be used in a larger portion of the stroke population, is more time- and cost-efficient, and can be reliably employed to delineate lesions (de Haan and Karnath, 2018; Mair and Wardlaw, 2014; Moore and Demeyere, 2022; Mullins et al., 2002; Vert et al., 2017). Large-scale studies have demonstrated that CT can identify established neural correlates of common post-stroke impairments (Gillebert et al., 2014; Moore et al., 2021; Moore and Demeyere, 2022). Despite these advantages, CT data have some key weaknesses. Mainly, hyperacute CTs have low sensitivity to ischemic infarcts compared to MR. Urbach et al. (2000) found that CT was 55% sensitive to ischemic stroke whilst diffusion weighted imaging was 100% sensitive within 6 hours of stroke. This issue decreases the proportion of CT scans that can be used for LSM but does not necessarily degrade the quality of data that can be collected from CT scans which do show visible lesions (de Haan and Karnath, 2018). Additionally, CT is thought to often fail to visualize the full extent of damage compared to MR (Bryan et al., 1991). Overall, it is commonly asserted that CT is inferior to MR within LSM. However, no quantitative analysis has been conducted to test this claim.

There are several key reasons why lesion-visualization differences between CT and MR scans may not significantly influence LSM results. First, hyper-acute CT scans may not visualize the full lesions extent, but stroke damage is generally clearly visible on later CT scans (Bryan et al., 1991). Bryan et al. (1991) found that lesions were visible on 58% of CT scans taken within 24 hours of stroke, but were visible in 88% of the same patients when CT scans were taken 7–10 days later. These later scans are generally used for LSM as they allow more confident segmentations (Moore, 2022). It is unclear whether these later CT scans yield lesion masks which differ significantly from MR-delineated lesions. Second, LSM generally employs non-linear transformations to warp native-space lesion masks into standard space templates (Brett et al., 2001; de Haan and Karnath, 2018; Rorden et al., 2012). This generally results in a significant loss of detail in original lesion maps, which could potentially negate differences present between native space MR and CT lesion masks (Brett et al., 2001). Finally, LSM results are subject to spatial mislocation due to non-random spatial variation patterns caused by the brain's vasculature structure and the inherent network structure of many behaviors of interest (Mah et al., 2014). These sources of bias are present within all LSM samples, regardless of imaging modality (Mah et al., 2014). Overall, it is unclear whether any variation due to input imaging modality is significant compared to these other common non-random sources.

The purpose of the present study is to conduct a quantitative comparison of the performance of CT- and MR-delineated lesions within LSM. First, this investigation aims to quantify agreement between lesion masks derived from CT and MR collected from the same patient. Next, these scan pairs are employed to evaluate the comparative performance of simulated LSM analyses employing exclusively CT or MR data. Finally, the results of these simulations are employed to identify factors underlying systematic differences in cluster displacement and overlap agreement. Overall, the findings of this study provide novel insight into the relationship between input modality and LSM reliability. This in turn will help guide future LSM studies towards producing reliable and generalizable results.

## 2 Methods and materials

### 2.1 Neuroimaging data

This study employs routine clinical neuroimaging data from patients recruited from the Oxford Cognitive Screen Program (NHS REC reference 14/LO/0648, 18/SC/0550, and 12/WM/00335) (Demeyere et al., 2015, 2019) and from patients recruited in Belgium (Ethics Committee Research UZ/KU Leuven S60062). All patients provided informed consent in line with the Declaration of Helsinki. Participants were considered for inclusion if they had available CT and MR (T2 MRI) data collected within the acute phase post-stroke which demonstrated visible, stroke-related lesions. Patients with clear evidence of multiple, temporally distinct strokes were excluded. All MR scans were T2 MRIs which are referred to as MR for brevity. Notably, although diffusion weighted imaging (DWI) is highly effective for identifying lesioned tissue (Urbach et al., 2000), DWI data were not available as it is not routinely collected in many clinical settings.

UK patients were selected from a database of routine clinical imaging containing 3034 scans collected from 1517 stroke patients. This database contains 847 patients with at least one useable CT scan and 147 patients with at least one useable MR scan. Of these patients, 110 first-time stroke patients had available CT and MR imaging collected within the acute stage (<31 days post-stroke). Within this sample, 19.1% (21) had no visible lesions on CT or MR scans, 17.3% had visible lesions on MR but not CT scans, and 2.7% had lesions visible on CT but not on MR. Four of the 67 patients with visible lesions on both CT and MR were excluded as their MR imaging was unable to be normalized. Overall, data from 85 patients (63/22 from UK/Belgium; average age = 67.4 [SD = 14.9, range = 26–89]; 44.7% female) were included. Scans were acquired a mean of 2 days following infarct (SD = 2.96, range = 0–21) with a mean time between CT/MR scans of 2.43 days (SD = 3.00, range = 0–21). CT scans were collected prior to MR in 72.3% of cases. 35 patients had left hemisphere lesions, 46 patients had right hemisphere lesions, and 4 lesions crossed the midline. Overall, 76 patients exhibited ischemic infarcts while 9 had hemorrhagic strokes. Notably, this study's sample size is large compared to the size of many LSM investigations. For example, a recent systematic review of 34 LSM studies found that the average sample size was 60 (range = 25–573) with 22/34 studies including less than 85 patients (Moore et al., 2023). In studies restricted to a single hemisphere, the average sample size was 75 (range = 25–203) with 8/25 studies including less than the 46 right hemisphere patients reported here (Moore et al., 2023). Notably, most (20/34) previous studies employed a combination of CT and MR (Moore et al., 2023).

Two binarized lesion masks were created for each patient, one using CT and one using MR. Lesions were manually delineated on the axial plane of native space, whole-brain scans using MRIcron (used on tablet or computer screens) (McCausland Centre for Brain Imaging, Columbia, SC, USA) by trained investigators (MJM, n = 63; HH, n = 22) blind to other modalities. Slice thickness varied between 0.625 and 5 mm. Lesion mask accuracy was confirmed by the most experienced rater (MJM) and by a trained radiologist. All lesion masks were smoothed at 5 mm full width at half maximum in the z-direction and binarized using a 0.5 threshold (Moore, 2022). This is a low level of spatial smoothing compared to many previous studies which employ 8–12 mm smoothed lesions (Stamatakis and Tyler, 2005). This smoothing level was selected in line with the standard pre-processing pipeline reported by Moore (2022). Lesion masks were then reoriented to the anterior commissure and warped into 1 × 1 × 1 mm stereotaxic space using Statistical Parametric Mapping 12 and Clinical Toolbox functions (Ashburner et al., 2016; Rorden et al., 2012) which use age-specific CT and MRI normalization templates (Rorden et al., 2012). All normalized lesions were visually inspected by trained investigators (MM, HH) for quality.

Importantly, this study uses routine clinical data to best represent the data that would be included in real-world LSM analyses (Bates et al., 2003; de Haan and Karnath, 2018). This approach improves generalizability, but introduces some variability. For example, CT and MR imaging were collected at different time points. This means that perfect lesion overlap cannot be expected to occur across modalities as these scans cannot be expected to visualize biologically identical lesions. Similarly, CT and MR imaging require different normalization procedures using different templates (Rorden et al., 2012). Given this inherent variability, it is important to emphasize that paired CT and MR images are not assumed to show the same lesions. Importantly, the aim of this project is not to evaluate whether CT and MR imaging produces similar lesion maps, but is instead to determine whether LSM analyses derived from these masks differ in accuracy.

## 3 LSM simulation

To quantitatively evaluate LSM result accuracy, ground-truth correlates must be known. This presents an issue for real-world LSM, as the exact correlates underlying a deficit are ultimately unknown. LSM simulations present a solution to this problem. In these analyses, behavioral scores are simulated from lesion data by defining a “critical lesion site” (Mah et al., 2014). If this critical site is a cluster of voxels (e.g., a ROI), behavioral scores can be represented by the percentage of this target cluster overlapping each lesion. When single voxels are used as critical sites (e.g., Mah et al., 2014), each patient whose lesion overlaps with the critical voxel is classed as “impaired” while all other patients are “unimpaired”. LSM analysis is then conducted using this simulated behavior, allowing for the agreement between LSM results and underlying ground-truth to be quantified (Mah et al., 2014). This approach has been used to investigate how spatial variation may bias lesion-mapping results (Mah et al., 2014), but has not been applied to investigating differences between scan modalities.

This simulation analysis was first performed on an ROI scale. Behavioural scores were simulated by comparing lesions to the 96 cortical ROIs defined by the Harvard-Oxford Cortical Atlas (https://fsl.fmrib.ox.ac.uk/fsl/fslwiki/Atlases). This atlas was used as it is a common reference in LSM studies (Moore et al., 2023; Moore and Demeyere, 2022) and balances parcellation complexity and ease of anatomical interpretation. For each considered ROI, behaviour was simulated by calculating the percent of ROI voxels impacted by each lesion. For example, a lesion overlapping with 60% of the angular gyrus would be assigned a score of 60 for an analysis that considered this region as the “target ROI”. Behavioural data was simulated for CT and MR independently and 96 theory-blind, voxel-wise lesion-mapping analyses were run within each data set. These analyses employed identical inclusion and control parameters (e.g., control for lesion volume, minimum overlap inclusion threshold) and used one-tailed pooled-variance t-tests to evaluate voxel significance. To evaluate the impact of different statistical corrections, both Bonferroni and family-wise error rate permutation (2000 permutations) corrections were employed. Bonferroni corrections are commonly used in LSM but have been previously reported to be extremely conservative (Mirman et al., 2018). Past research has suggested that permutation corrections optimally balance false positive and false negative rates (Mirman et al., 2018). Significant voxels surviving each approach are reported and compared to determine whether simulation performance is comparable.

Next, voxel-level simulation was conducted. This approach is not clearly representative of real-world LSM as deficits would not be expected to be linked to damage to single voxels. However, this approach offers a powerful method for precisely quantifying results displacement (Mah et al., 2014). All voxels impacted in at least 8 patients were considered as “critical voxels”. This process was applied to CT and MR data, completely independently, and the results compared across modalities. LSM analysis was run on a theory-blind voxel-wise basis using a modified version of NiiStat (https://github.com/neurolabusc/NiiStat). The Liebermeister measure was used to evaluate significance. These voxel simulations employed Bonferroni corrections and controlled for lesion volume (Mirman et al., 2018).

## 4 Statistical analyses

First, descriptive analyses were conducted to compare lesion masks derived from MR and CT. The agreement between each pair of normalized scans was evaluated in terms of lesion volume and Dice coefficient. Dice similarity coefficient is a metric for evaluating the similarity between image segmentation maps that is commonly used to evaluate the agreement between brain tissue segmentations. The Dice coefficient is calculated by dividing the number of voxels in the overlap between binarized masks by the average of the number of voxels included in each mask. Importantly, this experiment does not employ standard thresholds for determining goodness of agreement (e.g. Landis and Koch, 1977), as these inherently arbitrary thresholds are not clearly applicable to cases where the maximum theoretically achievable score is well below 1.00 (as in this experiment). Additionally, degree of agreement between CT and MR lesion masks in this study should not be interpreted in the same way as (and compared to) inter-rater lesion delineation agreement or performance of automated lesion segmentation methods. This is because these methods make comparisons across the same lesion quantified using the same scan, while this study compares across two lesions illustrated by different scans at different timepoints. Therefore, it is important to emphasize that neither the CT nor MR mask/results can be considered to represent a “ground truth” and therefore variation between these masks is expected to occur. This voxel-wise similarity coefficient is a very strict measure of agreement and low voxel-wise similarity values do not necessarily imply low replicability of LSM results in more realistic scenarios.

Next, descriptive analyses were conducted to summarize the results of ROI-level simulations employing CT and MR data. In ROI simulations, “hits” were defined as cases in which the significant voxels yielded by a simulation analysis overlapped with the target ROI. Accuracy relative to the target ROI was evaluated by calculating the Dice coefficient of results versus the target. As many LSM analyses report results in terms of the location of the highest results z-score (peak voxels), we also report the degree of overlap between the peak voxels and target. The agreement between CT and MR results employing the same target ROI (e.g. CT and MR analyses with the angular gyrus as a target) was also evaluated using Dice coefficients.

Analogous metrics are reported to evaluate the performance of voxel-level simulation analyses. Specifically, the proportion of target hits (results clusters containing the target voxel) and distance between peak voxel clusters and the target voxel are reported for each analysis. Importantly, the distance between peak voxel location and underlying target voxels could be expected to vary as a function of the size of the peak voxel cluster. This is because as the size of results clusters increases, the average distance between each peak voxel may be displaced towards the center of this cluster rather than the underlying target voxel. To address this potential issue, additional analyses are conducted which control for cluster size by weighting displacement measures (e.g. distance from target) by the number of voxels in each cluster (Zhang et al., 2014). This approach standardizes displacement metrics by considering distance relative to cluster size rather than raw displacement as an accuracy measure. These control analyses are included alongside all comparisons evaluating the distance between peak voxel clusters. Each of these metrics are calculated and statistically compared for all simulated CT and MR analyses. To facilitate direct comparisons across these modalities, performance metrics were then compared across paired MR and CT analyses considering the same underlying target voxel.

Finally, each simulated single-voxel analysis CT z-map was then evaluated relative to the paired MR z-map by calculating Dice coefficients across a range of significance thresholds. This analysis was conducted because Bonferroni-corrected significance thresholds are determined by the number of voxels analyzed, meaning that CT and MR analyses employ different z-score significance thresholds (4.5919 vs 4.7869 respectively) due Bonferroni correction threshold's dependence on the number of voxels tested (22,358 CT vs 58,998 MR voxels analyzed). To assess whether comparable spatial information was present in CT-based LSM outputs compared with the MR-based outputs, despite these simple Bonferroni-based thresholds, a series of different z-score thresholds were applied to facilitate comparisons that were then independent of mild-moderate differences in statistical power. For these analyses, the significance threshold for the MR maps was held constant at the Bonferroni-corrected z-score threshold whilst the paired CT threshold was varied across 50 evenly spaced values between 3 and the MR-based Bonferroni threshold. These data were used to identify the CT significance threshold that yielded the highest Dice coefficient. Importantly, perfect results overlap (Dice coefficient = 1) cannot reasonably be expected to occur for these results maps since neither the CT or MR results map can be expected to represent the “ground truth” of 1 significant voxel (Mah et al., 2014). This is largely because the compared scans are not collected at the same time point, so the displayed lesion will not be biologically identical across scans. This difference will result in variation across all conducted analyses. Additionally, systematic differences in results cluster size will constrain the maximum achievable agreement between CT and MR scans. For example, if a MR analysis yield twice as many significant voxels as the paired CT analysis, the maximum achievable Dice will be 0.66. For this reason, all reported Dice scores are interpreted relative to the maximum achievable overlap rather than to the perfect, unattainable Dice score of 1 (Landis and Koch, 1977).

## 5 Results

### 5.1 CT/MR lesion comparison analyses

**Fig. 1:**
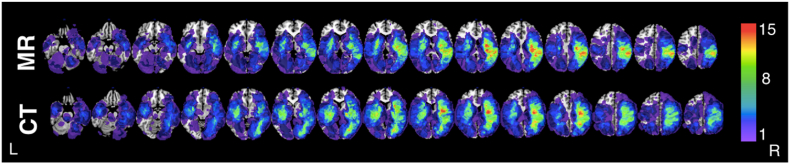
Comparative Lesion Overlays Comparative lesion overlays for the CT and MR data employed in this investigation. Voxel color denotes number of affected patients with voxels damaged in at least 8 patients being included in the reported simulation analyses.

**Fig. 2:**
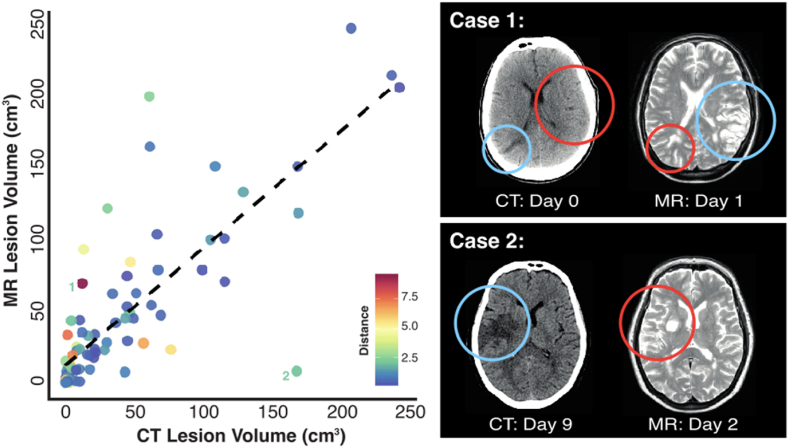
Agreement between CT- and MR-derived Lesion Masks A visualization of the relationship between the volume of paired lesion masks delineated on CT and MR images. Two outlier points (labeled 1 and 2) are illustrated in detail. In these charts, blue circles denote regions that were successfully delineated while red circles highlight areas that were missed. Case 1 represents a patient with two distinct lesions in which the CT and MR delineations each yielded a false negative and a true positive result, resulting in a large difference in lesion centers. In Case 2, an earlier MR scan failed to visualize the full extent/expansion of impacted tissue volume that was clearly visible on a later CT scan. Importantly, data were still included in other analyses to accurately represent errors/variation occurring in real-world LSM analyses.

First, lesion masks drawn from MR and CT data were compared (Fig. 1). The volume of MR lesions was larger than that of CT lesions, but this difference was not significant in a (paired *t*-test) (42.5 cm^3^ (median = 22.16, range = 0.14–239.14) versus 34.8 cm^3^ (median = 13.47, range = 0.11–241.15) respectively (paired Wilcoxon Sign-Ranked Test, V = 2086, p = 0.2583, 95% CI: −1.287–5.449). A Pearson Correlation revealed that volume for CT- and MR lesions was strongly correlated (correlation coefficient = 0.778, t(83) = 11.291, p < 0.001, CI: 0.678–0.850) (Fig. 2).

**Fig. 3:**
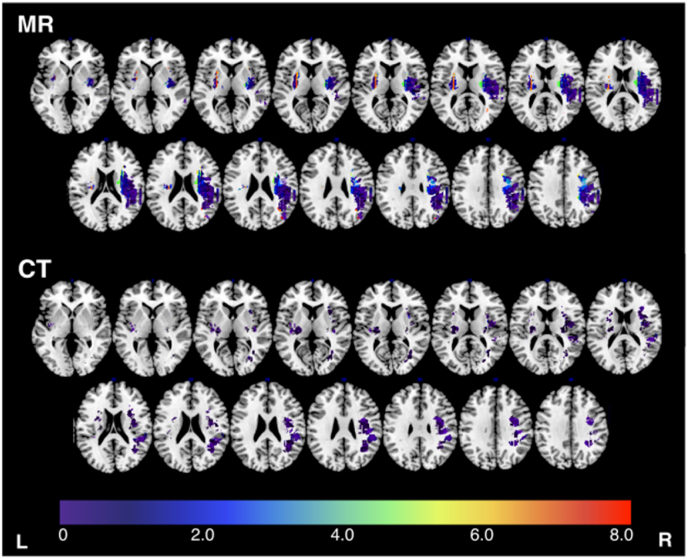
Target distance for CT and MR analyses.

**Fig. 4:**
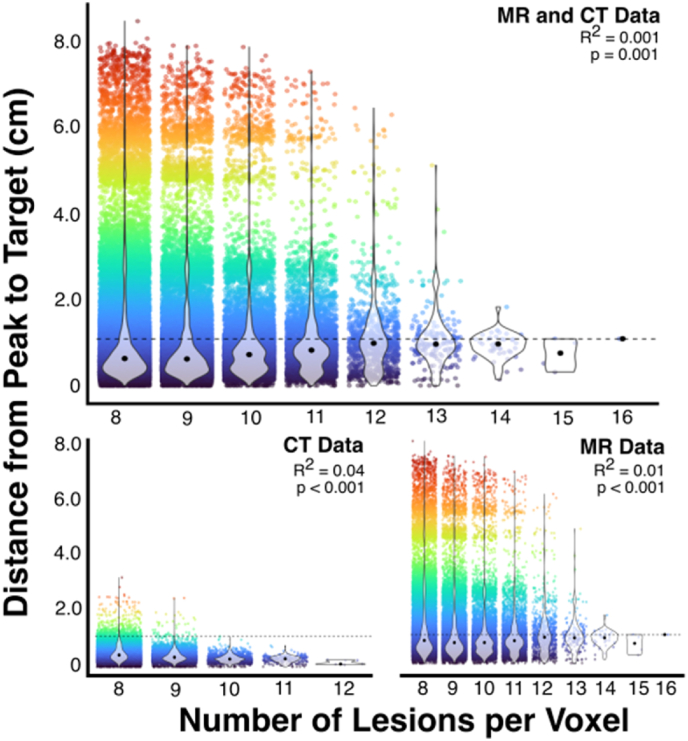
Relationship between number of lesions per voxel and distance from target A visualization of the relationship between the number of lesions that overlap with each considered target voxel and the distance between the peak cluster and this target. The horizontal line denotes the mean distance from target. Critically, this figure illustrates that clusters are closer to the target when more lesions impact the target voxel. Voxels impacted by a minimum of 8 lesions are included in this figure, as voxels overlapping with fewer lesions were not included in analysis.

**Fig. 5:**
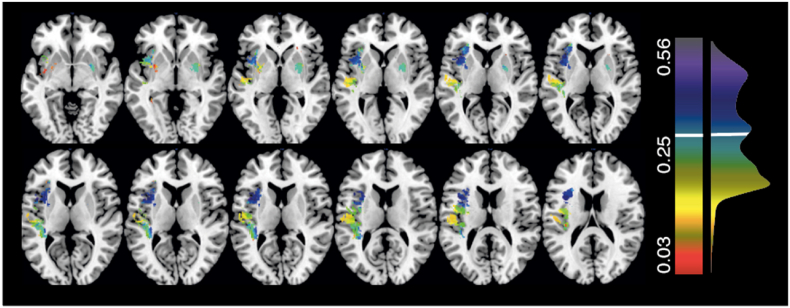
Dice coefficients for each analyzed target voxel A visualization of Dice coefficient of CT- and MR-derived results maps across different target voxel locations. All optimally-thresholded Dice coefficients within three standard deviations of the mean (0.340) are included in this visualization. The distribution of these Dice coefficients is represented to the right of the color scale, with the mean value marked in white.

**Fig. 6:**
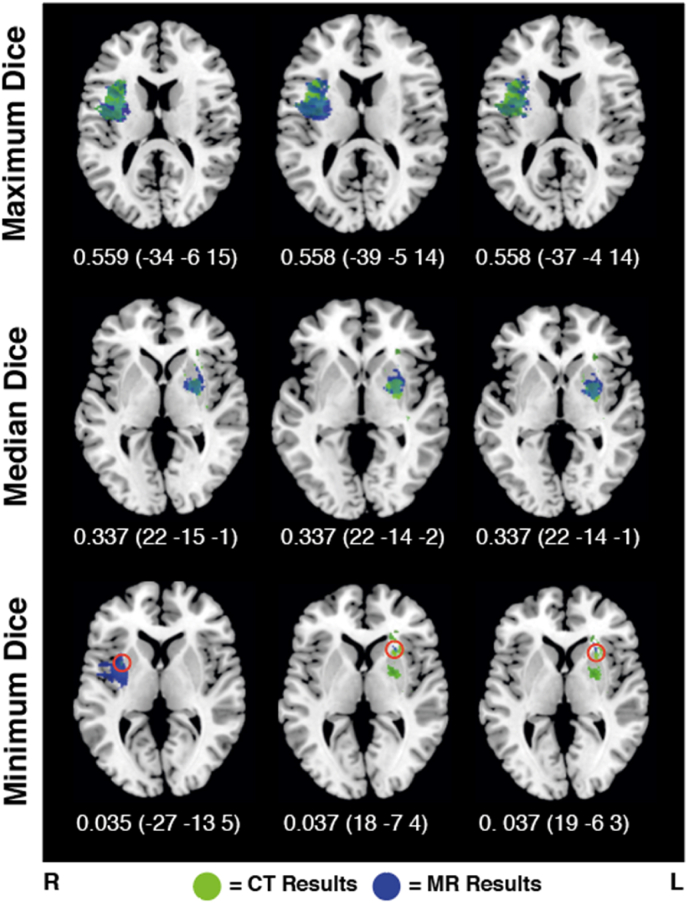
Example CT- and MR-derived Results Overlay Examples of the overlap between optimally thresholded results clusters yielded by paired MR/CT LSM analyses. The top row visualizes the three highest, non-outlier Dice coefficients, the middle row contains examples of the median Dice coefficient, and the lower row presents the three lowest non-outlier Dice coefficient examples. Each image presents the axial slice containing the target voxel, the Dice coefficient, and the MNI coordinates of the target voxel. In each example, the target voxel is located within the area of overlap between the CT and MR results maps (highlighted in red in the lower row).

These lesion pairs, however, often substantially differed in terms of overlap. This difference is at least in part due to the biological differences in lesions displayed on CT vs MR due to differences in scan collection times. Despite this inherent difference, the areas delineated on paired MR and CT scans had an average overlap of 30.4% (SD = 22.5%, range = 0–70.6%), with 29.9% (SD = 23.5%, range = 0.3%–95.6%) of all voxels marked as impaired in either CT or MR scans being marked as impaired only on CT scans and 39.6% (SD = 28.2%, range = 0.09–97.5%) being marked as impaired on only MR scans. This overlap yielded an average Dice coefficient of 0.420 (SD = 0.275, range = 0–0.828).

The largest difference in lesions reported by CT and MR was in a case where false negative delineation errors had been made on both MR and CT (Fig. 2, Case 1). Specifically, this patient exhibited two lesions, one of which was successfully delineated on the CT scan and the other of which was delineated on the MR. This is an important case to highlight as it demonstrates that both CT and MR scans can produce false negatives. This data was included in LSM simulations to accurately represent noise that would be present in real-world LSM investigations with access to only one imaging modality. Days between scans and days between stroke and scan collection were not found to act as significant predictors of similarity between the lesions displayed on CT versus MR quantified in terms of volume difference (F(2,71) = 1.247, adjusted R^2^ = 0.006, p = 0.294) or Dice coefficient (F(2,71) = 0.039 adjusted R^2^ = −0.027, p = 0.961).

## 6 ROI-level LSM simulation analyses

Within the ROI analyses, 62/96 ROIs met inclusion criteria within CT data and 93/96 ROIs met analysis inclusion criteria within MR data. Within these included ROIs, only voxels which were impacted in at least 8 patients were analyzed. In CT analyses, this criterion allowed only a mean of 19.7% (SD = 30.2, 0.01–100) of ROI voxels whilst MR could test 59.8% (SD = 32.3, Range = 0.15–100) of ROI voxels. ROI statistics are available in supplementary materials.

**Fig. 7:**
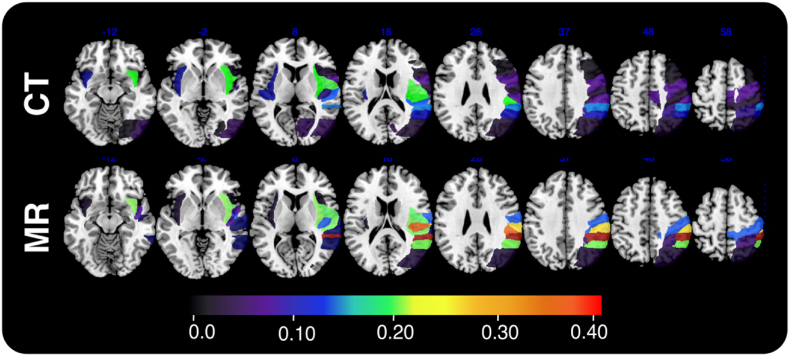
Visualization of the Dice coefficient of simulated CT and MR ROI-level analyses (Bonferroni corrected). ROI color represents Dice coefficient of the relevant analysis results versus the underlying target voxel. Only ROIs that were successfully identified by the relevant analyses are included in this visualization. Overall, no significant differences in CT-versus MR-derived analyses were identified.

In Bonferroni-corrected ROI simulations, significant voxels yielded by CT analyses overlapped with the target ROI in 22/62 analyses while MR-analyses overlapped with the target ROI in 17/93 analyses, with a significantly higher portion (X^2^ = 5.843, p = 0.156) of CT analyses identifying the target (Fig. 7). When compared to the underlying target ROI, CT results had a mean Dice coefficient of 0.013 (SD = 0.04, range = 0–0.21) whilst MR results had a mean Dice of 0.024 (SD = 0.07, range = 0–0.40), (t(153.98) = −1.17, p = 0.245, 95% CI: −0.027 – 0.007) (Fig. 7). This very low overlap between analyses and targets is partially due to the low lesion coverage of target ROIs. In cases where CT and MR analyses considered the same underlying target ROI, the average Dice coefficient of CT results versus MR results was found to be 0.038 (SD = 0.07, range = 0–0.26). In the 48 CT analyses yielding significant results, 19.1% of the peak z-score voxels overlapped with the target ROI. In the 31 MR analyses yielding significant results, 38.6% of peak voxels overlapped with the target.

Significant voxel clusters in both CT and MR analyses were significantly smaller than the target ROIs (CT: t(95) = −9.39, p < 0.001, CI: −162.0 -105.4 (cm^3^)) (MR: t(95) = −7.915, p < 0.001, CI: −154.2 to −92.4 (cm^3^)), and CT analyses yielded significantly fewer significant voxels than MR analyses (t(95) = −2.44, p = 0.016, CI = −18.9–1.94 (cm^3^)). The percentage of each ROI meeting testing inclusion criteria (e.g. impacted in at least 8 lesions) in LSM analysis did not significantly predict Dice coefficients within both the CT (F(1,94) = 0.065, p = 0.799, Adjusted R2 = −0.09) and MR (F(1,94) = 0.677, p = 0.413, Adjusted R2 = −0.003). Similarly, target ROI size did not predict Dice coefficients (CT: F(1,94) = 0.009, p = 0.350, Adjusted R2 = −0.001) (MR: F(1,94) = 0.589, p = 0.453, Adjusted R2 = −0.005).

When permutation-based statistical corrections were applied, CT and MR results overlapped with the target ROIs in 22/96 and 14/96 simulated analyses respectively. CT and MR analyses did not significantly differ in overlap with the target ROI (CT mean = 0.006, MR mean = 0.006, t(185.1) = −0.04, p = 0.9644, CI: −0.006 – 0.006). Peak voxels yielded by CT analyses had a higher overlap with target ROI than MR analyses (CT mean = 9.89% overlap, MR mean = 0.00% overlap, t(91) = 3.83, p < 0.001, CI: 0.05–0.151).

No significant difference was present in Dice coefficient between results and the target ROIs across Bonferroni and permutation corrections in CT analyses (t(134.2) = −1.54, p = 0.124, CI: −0.2–0.002). However, the Dice coefficient of MR analyses was significantly lower in permutation versus Bonferroni corrected analyses (t(113.3) = −2.234, p = 0.027, CI: −0.03 to −0.002). Similarly, the degree of overlap between peak voxels and target ROIs did not differ across Bonferroni and permutation-corrected results in CT analyses (t(77.43) = −1.72, p = 0.089, CI: −0.20 – 0.01), but was significantly higher in Bonferroni versus permutation corrections in MR analyses (t(30) = -4.655, p < 0.001, CI: 0.56 to −0.22).

## 7 Single-voxel level LSM simulation analyses

Next, voxel-level LSM simulation analyses were conducted. Within MR data, 58,998 voxels were lesioned in at least 8 patients, yielding 58,998 simulated LSM analyses with a Bonferroni-corrected z-score threshold of 4.7869. These analyses yielded an average of 12,214.6 (SD = 7021, range = 35–26,716) significant voxels. MR analyses yielded a mean of 196 peak z-score voxels per analysis (range = 1–1818, SD = 270.3) which were a mean distance of 1.39 cm (SD = 1.43) from the target voxel.

Within CT, 22,358 simulated LSM analyses were conducted (Bonferroni-corrected z-score significance threshold = 4.5919). These analyses yielded an average of 3688.8 (SD = 1635.2, range = 73–7826) significant voxels. The average distance between the center of mass of the significant voxel and the target voxel was 0.76 cm (SD = 0.38, range = 0–8.89 cm) (Fig. 3). CT analyses yielded a mean of 211 peak z-score voxels per analysis (range = 1–1438, SD = 350.5) which were a mean distance of 0.440 cm (SD = 2.96) from the relevant target voxel.

CT analyses yielded results clusters which were significantly closer to the underlying target voxel than MR analyses when this distance was quantified in terms of distance between peak voxels and target (t(60,810) = −141.4, p < 0.001, CI = −9.45 to −9.19). This difference is unlikely to be fully accounted for by differences in the size of results clusters as this result remained significant when results cluster size was controlled for (t(58,829) = −49.75, p < 0.001, CI: −0.96 to −0.89).

A visualization of the distance between the peak voxel cluster and the corresponding target voxel for all simulated LSM analyses. Each considered target voxel is color coded according to its distance (cm) to the corresponding results cluster. Notably, MR-based LSM analyses were able to test substantially more voxels, but were not necessarily closer to the target voxel than CT-based LSM analyses.

There were 14,135 voxels analyzed within both the CT and MR simulations. Within these voxels, There was no significant difference in the average distance between peak voxels and the target voxel between CT (mean distance = 0.457 cm (SD = 0.27) and MR (mean distance = 0.452 (SD = 0.29)) analyses (t(14,135) = 1.80, p = 0.072, CI: −0.005 – 0.110). When differences in results/peak voxel clusters were controlled for, CT analyses yielded results which were closer to the target voxel in terms of peak voxel distance (t(13,578) = −10.3, p < 0.001, CI: −0.07 – 0.05). Distance between each peak cluster and the underlying target was significantly associated with the number of lesions that overlapped with each location (F(81,354, 1) = 10.69, p = 0.001, Adjusted R^2^ = 0.001) (Fig. 4). The average distance between peak voxels and target voxels was higher (less accurate) when higher numbers of peak voxels were yielded by analyses (F(1, 27,156) = 1102, p < 0.001, adjusted R2 = 0.288.

## 8 Comparison of MR/CT single-voxel level LSM Z-maps

Next, the degree of agreement between the 14,135 paired CT and MR z-maps was evaluated across a range of significance cutoffs. In this analysis, the MR z-map was binarized according to the Bonferroni-corrected z-score cutoff whilst the CT z-map was binarized according to 50 evenly spaced z-score cutoffs (range = 3–4.7869). Across all considered thresholds, the average Dice coefficient between CT and MR was found to be 0.335 (SD = 0.103, range = 0–0.559). The maximum average Dice coefficient of 0.340 was achieved when the CT z-score significance threshold was set to 3.693. The average Dice coefficient was not heavily influenced by the choice of threshold (Dice range across all considered significance thresholds = 0.322–0.340, SD = 0.005). The Dice coefficient at this optimal z-cut threshold was found to be significantly higher than the Dice coefficient at the original, Bonferroni-corrected z-cutoff (t(27,081) = 14.942, p < 0.001, CI: 0.016–0.021), although this effect was small (Cohen's d = 0.18). Fig. 5 provides a visualization of how the Dice coefficient was found to vary with the location of the underlying target voxel. Fig. 6 provides an illustration of the overlap between CT and MR results maps at different Dice coefficient values.

Notably, a perfect Dice coefficient of 1 can only be achieved when both compared segmentation masks are exactly the same, and thus are the same size. As MR analyses were able to test approximately twice as many voxels as CT analyses, the resulting voxel clusters would not be expected to be comparable in size. In line with this, MR comparisons were found to yield significantly and substantially larger results clusters than CT analyses (t(15,713) = −94.06, p < 0.001, CI: −593.98 to −5710.88, Cohen's d = 1.14). For this reason, if the optimally thresholded LSM results clusters were maximally overlapping, the maximum achievable average Dice coefficient is 0.72 (SD = 0.17, range = 0.016–1). This value is derived from comparing the volume of results clusters across each matched CT and MR analysis, and determining what the maximum achievable Dice would be if these clusters exhibited the best possible degree of overlap. The actual achieved Dice coefficient value (0.34) must be evaluated in the context of this value to be effectively interpreted.

## 9 Discussion

The results of this study suggest CT and MR-derived lesion masks do not reliably differ in accuracy within LSM analyses. Although there are differences in the results maps yielded by CT and MR analyses, both CT and MR-derived analyses yielded largely overlapping results which both identified the underlying neural target with a similar degree of accuracy (Fig. 6). Importantly, both CT and MR analyses varied in accuracy as a function of lesion overlap, preforming poorly in cases where target areas had low statistical power. Given these mixed results, this study did not identify strong evidence that MR and CT imaging preform differently in LSM analyses (in terms of accuracy). Critically, this project does not aim to assert that one imaging modality is superior to another in LSM. The best imaging modality for real-world LSM studies depends on additional factors including scan availability, time of imaging, and imaging quality. However, the findings of this study suggest that both CT and MR imaging can potentially be used in LSM.

Both CT and MR analyses performed comparatively poorly (mean dice <0.25) in the ROI-level analyses, but CT was found to significantly outperform MR in terms of proportion of results overlapping with target ROIs. The number of lesions overlapping with each target voxel was found to be a key predictor of displacement magnitude, with higher lesion overlays producing results that were closer to the underlying target. The results yielded by paired voxel-level CT and MR LSM analyses were not systematically different in terms of Dice coefficients when systematic differences in cluster size and lesion overlay are considered.

## 10 The relationship between MR- and CT-delineated lesion masks

No significant systematic difference in lesion size between paired CT and MR scans was detected, and the volume of the lesion masks was highly correlated. Cases in which the located lesions differed greatly were explained by false negative delineations on both CT and MR masks. Paired CT- and MR-delineated lesions were found to have an average Dice coefficient of 0.42. This Dice coefficient may initially seem to be low, but there are several sources of unavoidable variation in this data set, which account for this difference.

The reasons for the low Dice coefficient include the fact that CT and MR scans were collected at different time points. Whilst these scans were generally collected within 2 days of stroke, temporal evolution of lesions occurs within hours (Urbach et al., 2000). This temporal variation can impact the lesion boundaries that are able to be delineated on both CT and MR scans, leading to disagreement between the delineated lesions. Additionally, the individual cases highlighted in this investigation also illustrate that neither CT nor MR delineated lesions should necessarily be considered as ground-truth measurements, as both CT and MR scans yielded false negative results and were susceptible to underestimating the full extent of lesion damage (see Fig. 2). Next, all lesion masks underwent non-linear spatial normalization to facilitate direct comparisons. Spatial normalization is an essential pre-processing step, but non-linear transformations result in some degree of distortion (e.g. interpolation-based distortion due to differences in slice thickness), which can be different between CT and MR (Brett et al., 2001; Rorden et al., 2012). Finally, previous research has demonstrated that Dice coefficients are reduced due to voxel-wise disagreements near the borders of small lesions, with larger lesions yielding higher Dice coefficients (De Haan et al., 2015; Trutti et al., 2021). For these reasons, perfect overlap should not be expected between the lesion pairs employed in this study.

## 11 LSM simulations

Within ROI analyses, both CT and MR analyses yielded very low Dice coefficients (<0.03). CT analyses yielded a significantly higher proportion of results which overlapped with the true target cluster than matched MR analyses. Specifically, 35.5% of CT analyses overlapped with the target versus 18.3% of MR analyses. There are several factors that may account for this generally poor performance. First, not all voxels within each target ROI have sufficient lesion coverage. For this reason, power varies within each included ROI and many voxel-wise false negatives may have resulted from insufficient lesion overlap. LSM is unable to disambiguate between the role of damage to ROIs that are commonly damaged together (Mah et al., 2014). For example, as MCA strokes generally damage both the insular cortex and the neighboring opercular cortices, the results of analyses considering either of these ROIs as targets would be expected to be erroneously enlarged to include these neighboring areas (Mah et al., 2014). This effect will result in reduced dice coefficients as these “false positive” areas will not be consistent in location and extent across CT and MR results. Importantly, these issues are inherent in all LSM analyses, regardless of imaging modality. Although CT analyses were found to outperform MR analyses in terms of proportion of results overlapping with the target ROI, CT and MR analyses exhibited no difference in Dice coefficient versus the underlying target. This lack of difference may potentially be attributed to floor effects, as both imaging modalities yielded very low Dice coefficients relative to the target ROI. Overall, the results of the ROI-level LSM analyses highlight established shortcomings in traditional univariate LSM methodology, but also suggest that both CT and MR analyses are similarly impacted by these issues.

Within voxel simulations, MR peak voxels were an average of 1.39 cm from the target voxel whilst CT peaks were a mean of 0.44 cm from the target. Previous research has suggested that LSM clusters are generally displaced by approximately 1.5 cm due to inherent, non-random spatial lesion distributions (Mah et al., 2014). Compared to this value, the analyses in this study generally performed well in terms of cluster location relative to the underlying target. In this study MR-based analyses were able to test more voxels than CT-based analyses. However, in real-world analyses, CT data is generally more widely available than MR data. For example, our database of routinely collected stroke neuroimaging includes scans from 1517 patients with 847 (55.8%) having CT and 147 (9.7%) having MR scans which can be used for LSM. This high proportion of unusable scans is not only due to lesions not being visible as patients with multiple stroke events (estimated 17% of population (Baird et al., 2000), 19% within our own database), severe atrophy (which precludes spatial normalization), and poor scan quality (e.g. movement artifacts) must also be excluded from LSM analyses. As the number of testable voxels generally increases as the number of patients included rises, this difference in comparative availability implies that real-world studies using CT data would likely be able to test more voxels than analyses that only include MR. Taken together, the results of this study suggest that CT-delineated lesion masks yield results of similar accuracy to those produced by MR-based lesion masks. Specifically, MR and CT results produced results clusters which varied in size and degree of overlap, but exhibited clear qualitative agreement in terms of accuracy relative to the underlying target (illustrated in Fig. 6). There are several potential explanations for this result.

MR analyses yielded larger significant voxel clusters than CT analyses. There was a significant relationship between cluster size and displacement in the simulation results, with smaller clusters having peaks that are nearer to the underlying target voxel compared to larger clusters. This effect likely underlies a portion of the systematic difference in displacement distance identified between CT-based and MR-based analyses. However, this difference cannot be fully accounted for by differences in the volume of peak voxel clusters yielded by MR and CT analyses, as displacement differences remained significant when the volume of peak clusters was included as a covariate. Additionally, CT analyses yielded more peak voxels than MR analyses, but these CT peaks were closer to the underlying target than those yielded by MR. Importantly, the identified relationship between cluster size and peak displacement is likely to be specific to the simulation methodology and would not necessarily be expected to impact the results of real-world analyses. However, this effect somewhat complicates the interpretation of cluster displacement relative to target voxels as a metric for comparing the performance of CT- and MR-based LSM analyses. Future research is needed to examine whether the documented differences between CT and MR-based lesion mapping analyses are maintained when the number and location of included voxels is more similar across modality groups. More informative conclusions can be drawn by evaluating the overlap between the clusters derived from CT and MR analyses that employed the same underlying target voxel, as these matched comparisons quantify how imaging modality influences the results of analyses considering identical targets.

There was a moderate degree of agreement between the clusters produced by CT and MR analyses employing the same target. The achieved agreement between paired MR and CT results would not have been possible if the CT results maps did not contain similar information to MR masks. Given the size difference between MR and CT LSM results clusters, the highest possible achievable average Dice coefficient was 0.72 (derived by calculating the maximum possible overlap of each paired MR/CT results cluster). Considering this alongside the expected variation due to different lesion inputs, different lesion overlays, and differences in LSM cluster sizes, the actual achieved Dice coefficient of 0.34 can be considered to be indicative of moderate agreement. This conclusion is supported by the qualitative overlap between CT and MR results (illustrated in Fig. 6), which demonstrate that despite the apparently low Dice coefficient, these results clusters are reporting the same underlying neural region. This illustrates that despite differences in exact overlap, MR- and CT-derived lesion masks will likely lead to similar anatomical conclusions in real-world LSM analyses.

Importantly, the number of lesions overlapping with each target voxel was found to predict analysis accuracy. In both the CT and MR voxel-level simulations, the higher the lesion overlap was with the target voxel, the closer the cluster center was to the target. This effect is likely driven by the fact that lower overlaps will occur more at the edge of a common territory and the increase in statistical power, with the associated reduction in false negatives, when the tested voxel is damaged in more patients. This effect was present despite the large number of lesions included in these studies and the comparatively high lesion overlap inclusion threshold (minimum n = 8 compared to the commonly used minimum n = 4 (Mah et al., 2014)) Real-world LSM analyses generally test voxels damaged in at least 5–10% of the included sample, but many LSM analyses employ small samples (n < 25) (de Haan and Karnath, 2018; Moore et al., 2021). The present study suggests that this may lead to decreased precision and generalizability of results. Notably, degree of lesion overlap is not only related to the number of patients in the study but is primarily determined by the spatial distribution of lesions. For this reason, it is critically important for future LSM analyses to include not only a high number of patients but also a diverse range of lesions to facilitate reliable and generalizable inferences.

Importantly, there are additional that future investigations should consider before deciding on LSM imaging modality. It generally requires a higher level of expertise to delineate acute stroke lesions on CT versus MR scans (Bryan et al., 1991; Urbach et al., 2000). It is also important to consider the time interval between stroke, scan collection, and behavioral assessment. For example, if CT scans are collected in the very early stages post-stroke (e.g. <6 hours) a smaller proportion will be useable for LSM. Alternatively, research MR scans collected well after the stroke event or behavioral testing (e.g. >3 months) are also not suitable for many LSM analyses (de Haan and Karnath, 2018; Karnath and Rennig, 2017). LSM studies should aim to include behavioral and neuroimaging data collected at similar timepoints (e.g. within one week) as soon as possible following stroke (e.g. < 1 month) (de Haan and Karnath, 2018; Karnath and Rennig, 2017). Overall, the best imaging choice for any given study is dependent on imaging quality, timing, and availability within the target sample.

Considered cumulatively, the results of this study demonstrate that CT and MR-derived lesion masks do not reliably differ in accuracy across scan modalities Although there are differences present in the results maps yielded by CT and MR analyses, both CT and MR-derived analyses yielded overlapping results which both identified the underlying neural target (Fig. 6).

These findings support de Haan and Karnath's (2018) assertion that LSM can use CT-derived lesion masks. Hyper-acute CT scans do have a high lesion false-negative compared to MR, but this does not necessarily mean the quality of data that can be collected from later CT scans is reduced, as these do show visible lesions. CT scans are generally more available than MR scans (Mair and Wardlaw, 2014; Singer et al., 2004), meaning that studies employing CT data will allow larger and more representative samples to be included in LSM analyses. Therefore, LSM analyses can aim to utilize CT scans, as excluding patients without MR scans risks introducing severe sampling biases (de Haan and Karnath, 2018; Singer et al., 2004). Previous work has concluded that investigations can also include a combination of CT and MR data to improve the size and representativeness of their patient samples (de Haan and Karnath, 2018). This increased sample size will, in turn, increase the number of lesions overlapping with each tested voxel, which was demonstrated to decrease the displacement between LSM clusters and the underlying location of interest.

## 12 Limitations

This investigation employed simulated behavior to facilitate quantitative measurements of the spatial maps associated with a single target. In real-world LSM, underlying regions causing deficit(s) can either be a spatially contiguous voxel cluster or a diffuse network of connected, but equally necessary components (Bartolomeo et al., 2007; Thiebaut de Schotten et al., 2011). This study's simulates behavior based using imaging units produced by scanners (voxels) which do not correspond to any real-world functional units. These simulations are “perfect” and do not include behavioral noise which would be encountered in real analyses. These analyses are not meant to replicate real-world LSM but instead aim to identify factors that bias results in an artificial, idealized scenario.

Moreover, as this is a retrospective study, CT and MR scans were not matched on all potentially relevant features (e.g., time of imaging). Similarly, smoothing may have a differential impact on MR and CT-derived lesions. As only patients with both image modalities were included the sample may not be fully representative of the stroke population (e.g., relatively few hemorrhagic strokes). Importantly, univariate LSM is an imperfect methodology. Recent research has suggested that multivariate or disconnection-based LSM may outperform traditional univariate approaches (Foulon et al., 2018; Mah et al., 2014; Zhang et al., 2014). However, univariate LSM is still commonly employed so it remains important to identify how neuroimaging modality may impact results.

## 13 Conclusions

Overall, the results of this study suggest that LSM analyses based on CT and MR input data produce results do not reliably differ in accuracy, even though numerous differences exist in the statistical maps. This finding is critically important in the context of real-world LSM analyses as it none of the results contradicted previous assertions that lesion-mapping analyses can employ CT-derived lesion masks if these scans are available within the appropriate time-window (de Haan and Karnath, 2018). The results of this study also highlight the importance for future LSM analyses to aim to capture a wide variety of patients so that there is a high degree of overlap (e.g. >8 patients per voxel), as LSM results were found to be closer to the underlying target as the number of patients with lesions overlapping each target voxel increased. This overlap will increase as sample size increases, highlighting the importance of using large samples in LSM analyses. These practices, in turn, will help improve the quality of LSM analyses, as they will greatly increase the size and representativeness of samples included in LSM analyses. Taken together, the results of this study provide novel insight into the relationship between input modality and LSM performance and help guide researchers towards designing high-quality, informative LSM analyses.

## Credit author statement

**Margaret J. Moore**: conceptualization, investigation, Writing - original draft, writing review & editing methodology, data curation, formal analysis, visualization, **Mark Jenkinson**: conceptualization, methodology, formal analysis supervision, writing review & editing, **Ludovica Griffanti:** methodology, writing review & editing, **Hanne Huygelier:** investigation, data curation, writing review & editing, **Celine R. Gillebert:** investigation, resources, writing review & editing, supervision, funding acquisition, **Nele Demeyere:** conceptualization, resources, writing review & editing, supervision, project administration, funding acquisition.

## Funding

This work was funded by 10.13039/501100000364Stroke Association UK awards to ND (TSA2015_LECT02) and MJM (SA PGF 18\100031) and the 10.13039/501100003130Research Foundation Flanders (G0H7718N) to CRG and (1249923N) HH. MJ is supported by the 10.13039/100014461National Institute for Health Researc (NIHR) Oxford Biomedical Research Centre (BRC), and this research was funded by the 10.13039/100010269Wellcome Trust (215573/Z/19/Z). LG is supported by an 10.13039/501100000320Alzheimer's Association Grant (AARF-21-846,366) and the 10.13039/100014461NIHR Oxford Health Biomedical Research Centre. This work was also supported by the 10.13039/100010269Wellcome Centre for Integrative Neuroimaging, which has core funding from the 10.13039/100010269Wellcome Trust (203139/Z/16/Z). For the purpose of open access, the author has applied a CC-BY public copyright licence to any Author Accepted Manuscript version arising from this submission.

## Declaration of competing interest

The authors declare that they have no known competing financial interests or personal relationships that could have appeared to influence the work reported in this paper.

## Data Availability

All anonymised data is available at https://osf.io/ed8rz/
