# Supplementary material for: A comparison of lesion mapping analyses based on CT versus MR imaging in stroke
Source: Neuropsychologia. 2023 Jun 6;184:108564. doi: 10.1016/j.neuropsychologia.2023.108564 (PMC10933788; doi:10.1016/j.neuropsychologia.2023.108564)
Supplement: Multimedia component 1 [file mmc1.docx]

**Supplementary Materials:**

**Table 1:** Descriptive statistics for each of the ROIs considered within the ROI-level simulation. Fraction reports the average percent of the ROI damaged in each lesion. Extension reports the average percent of each lesion which impact the relevant ROI. Percent analyzed reports the percent of ROI-voxels which were impacted in at least 8 patients (e.g. the voxels able to be included lesion-mapping analysis). Data means are presented alongside standard deviations (in parentheses).

|  |  | **CT Data** | | |  | **MR Data** | | |
| --- | --- | --- | --- | --- | --- | --- | --- | --- |
| **ROI** | **Hem.** | **Fraction** | **Extension** | **% Analysed** |  | **Fraction** | **Extension** | **% Analysed** |
| Angular Gyrus | L | 2.35% (9.7) | 0.63% (3.9) | 54.24% |  | 1.92% (9.0) | 0.4% (2.9) | 96.25% |
| Central Opercular Cortex | L | 5.10% (18.8) | 0.66% (2.1) | 77.08% |  | 4.13% (17.0) | 0.62% l(1.8) | 99.99% |
| Cingulate Gyrus anterior division | L | 0.34% (2.9) | 0.1% (0.6) | 0.00% |  | 0.11% (1.0) | 0.05% (0.4) | 9.51% |
| Cingulate Gyrus posterior division | L | 0.35% (1.6) | 0.06% (0.3) | 0.00% |  | 0.33% (2.0) | 0.06% (0.5) | 42.66% |
| Cuneal Cortex | L | 2.94% (14.8) | 0.37% (1.8) | 0.00% |  | 3.61% (17.0) | 0.41% (2.0) | 52.08% |
| Frontal Medial Cortex | L | 0.01% (0.1) | 0% (0) | 0.00% |  | 0.01% (0) | 0% (0) | 0.00% |
| Frontal Operculum Cortex | L | 3.27% (11.8) | 0.21% (0.7) | 4.52% |  | 2.36% (10.0) | 0.18% (0.7) | 100.00% |
| Frontal Orbital Cortex | L | 1.11% (4.8) | 0.29% (1.2) | 0.25% |  | 0.83% (4.0) | 0.27% (1.3) | 59.39% |
| Frontal Pole | L | 0.38% (2.2) | 0.68% (4.7) | 0.00% |  | 0.22% (2.0) | 0.38% (3.5) | 16.29% |
| Heschl's Gyrus | L | 6.47% (23.3) | 0.23% (1.0) | 96.05% |  | 5.46% (21.0) | 0.26% (1.1) | 100.00% |
| Inferior Frontal Gyrus pars opercularis | L | 2.4% (11.8) | 0.16% (0.7) | 0.25% |  | 1.71% (10.0) | 0.14% (0.6) | 99.81% |
| Inferior Frontal Gyrus pars triangularis | L | 0.76% (2.6) | 0.14% (0.5) | 0.06% |  | 0.48% (2.0) | 0.09% (0.4) | 92.19% |
| Inferior Temporal Gyrus anterior division | L | 0.78% (4.6) | 0.05% (0.3) | 2.72% |  | 0.41% (3.0) | 0.01% (0.1) | 85.44% |
| Inferior Temporal Gyrus posterior division | L | 0.61% (3.2) | 0.13% (0.8) | 21.94% |  | 0.33% (2.0) | 0.04% (0.2) | 83.34% |
| Inferior Temporal Gyrus temporooccipital part | L | 0.19% (1.4) | 0.01% (0.1) | 11.38% |  | 0.3% (2.0) | 0.02% (0.1) | 56.56% |
| Insular Cortex | L | 4.98% (16.7) | 1.52% (4.4) | 63.35% |  | 4.27% (15.0) | 1.29% (3.4) | 99.94% |
| Intracalcarine Cortex | L | 1.77% (11.1) | 0.51% (3.5) | 1.20% |  | 2.6% (14.0) | 0.48% (3.1) | 71.65% |
| Juxtapositional Lobule Cortex | L | 1.97% (12.6) | 0.24% (1.7) | 0.00% |  | 1.02% (9.0) | 0.24% (2.6) | 34.51% |
| Lateral Occipital Cortex inferior division | L | 0.72% (4.4) | 0.27% (1.5) | 20.99% |  | 1.02% (7.0) | 0.24% (1.4) | 85.24% |
| Lateral Occipital Cortex superior division | L | 2.22% (10.0) | 1.44% (6.3) | 10.83% |  | 2.03% (10.0) | 1.2% (5.5) | 56.33% |
| Lingual Gyrus | L | 0.36% (2.2) | 0.05% (0.3) | 0.00% |  | 1.09% (7.0) | 0.29% (2.3) | 82.22% |
| Middle Frontal Gyrus | L | 1.19% (4.9) | 0.36% (1.4) | 0.00% |  | 0.77% (4.0) | 0.25% (1.1) | 49.28% |
| Middle Temporal Gyrus anterior division | L | 2.73% (13.4) | 0.2% (1.1) | 45.10% |  | 1.63% (10.0) | 0.08% (0.4) | 89.12% |
| Middle Temporal Gyrus posterior division | L | 2.4% (12.0) | 0.37% (1.8) | 67.99% |  | 1.64% (10.0) | 0.17% (0.9) | 97.10% |
| Middle Temporal Gyrus temporooccipital part | L | 1.52% (7.4) | 0.09% (0.4) | 78.92% |  | 1.51% (8.0) | 0.1% (0.5) | 98.74% |
| Occipital Fusiform Gyrus | L | 0.28% (2.2) | 0.03% (0.3) | 3.10% |  | 0.98% (8.0) | 0.1% (0.7) | 99.01% |
| Occipital Pole | L | 1.01% (5.1) | 0.28% (1.4) | 0.11% |  | 1.16% (7.0) | 0.35% (1.7) | 31.39% |
| Paracingulate Gyrus | L | 0.33% (1.8) | 0.08% (0.5) | 0.00% |  | 0.16% (1.0) | 0.07% (0.6) | 2.55% |
| Parahippocampal Gyrus anterior division | L | 0.07% (0.4) | 0.01% (0.0) | 0.47% |  | 0.07% (0) | 0.01% (0) | 20.30% |
| Parahippocampal Gyrus posterior division | L | 0.21% (1.0) | 0.04% (0.30) | 0.00% |  | 0.27% (1.0) | 0.04% (0.2) | 47.96% |
| Parietal Operculum Cortex | L | 3.82% (15.5) | 0.24% (1.0) | 100.00% |  | 3.72% (15.0) | 0.32% (1.1) | 100.00% |
| Planum Polare | L | 5.39% (21.1) | 0.29% (1.3) | 80.46% |  | 4.7% (20.0) | 0.28% (1.2) | 100.00% |
| Planum Temporale | L | 4.66% (17.6) | 0.31% (1.4) | 95.49% |  | 4.47% (18.0) | 0.39% (1.7) | 100.00% |
| Postcentral Gyrus | L | 1.43% (6.3) | 0.91% (5.0) | 22.36% |  | 1.28% (6.0) | 1.24% (6.6) | 58.65% |
| Precentral Gyrus | L | 2.09% (8.7) | 1.14% (3.7) | 3.46% |  | 1.41% (7.0) | 0.96% (3.6) | 56.34% |
| Precuneous Cortex | L | 1.36% (7.7) | 0.44% (2.6) | 0.07% |  | 1.74% (9.0) | 0.62% (3.3) | 39.97% |
| Subcallosal Cortex | L | 0% (0) | 0% (0) | 0.00% |  | 0% (0) | 0% (0) | 0.00% |
| Superior Frontal Gyrus | L | 1.59% (8.3) | 0.73% (4.3) | 0.00% |  | 0.67% (4.0) | 0.42% (2.7) | 11.14% |
| Superior Parietal Lobule | L | 1.28% (5.9) | 0.26% (1.2) | 4.62% |  | 0.84% (5.0) | 0.17% (0.9) | 52.59% |
| Superior Temporal Gyrus anterior division | L | 4.97% (19.7) | 0.22% (0.9) | 52.45% |  | 3.4% (16.0) | 0.13% (0.7) | 99.54% |
| Superior Temporal Gyrus posterior division | L | 3.3% (14.6) | 0.37% (1.7) | 80.05% |  | 2.82% (14.0) | 0.32% (1.6) | 99.99% |
| Supracalcarine Cortex | L | 2.3% (11.8) | 0.22% (1.1) | 0.78% |  | 3.33% (15.0) | 0.25% (1.2) | 58.21% |
| Supramarginal Gyrus anterior division | L | 1.39% (8.4) | 0.18% (0.9) | 60.34% |  | 1.42% (8.0) | 0.21% (1.0) | 96.23% |
| Supramarginal Gyrus posterior division | L | 1.38% (7.1) | 0.2% (0.8) | 58.72% |  | 1.44% (7.0) | 0.22% (1.0) | 98.41% |
| Temporal Fusiform Cortex, posterior division | L | 1.97% (11.1) | 0.13% (0.7) | 3.56% |  | 1.32% (9.0) | 0.08% (0.53) | 61.08% |
| Temporal Fusiform Cortex anterior division | L | 0.13% (1.0) | 0% (0) | 0.34% |  | 0.55% (5.0) | 0.01% (0.1) | 24.19% |
| Temporal Occipital Fusiform Cortex | L | 0.22% (1.3) | 0.02% (0.11) | 0.04% |  | 0.52% (3.0) | 0.04% (0.2) | 94.37% |
| Temporal Pole | L | 1.17% (6.0) | 0.3% (1.3) | 4.82% |  | 1% (6.0) | 0.22% (1.2) | 56.81% |
|  |  |  |  |  |  |  |  |  |
| Angular Gyrus | R | 4.38% (15.2) | 0.9% (2.8) | 0.00% |  | 5.16% (17.0) | 0.93% (2.8) | 40.82% |
| Central Opercular Cortex | R | 7.21% (18.3) | 0.88% (2.1) | 1.49% |  | 7.04% (18.0) | 0.92% (2.8) | 100.00% |
| Cingulate Gyrus anterior division | R | 0.66% (3.04) | 0.75% (5.9) | 0.00% |  | 0.47% (2.0) | 0.41% (4.3) | 6.38% |
| Cingulate Gyrus posterior division | R | 1.6% (6.9) | 0.15% (0.5) | 0.01% |  | 1.25% (6.0) | 0.11% (0.4) | 13.43% |
| Cuneal Cortex | R | 2.77% (9.0) | 0.68% (2.9) | 0.00% |  | 2.63% (9.0) | 0.57% (2.7) | 52.94% |
| Frontal Medial Cortex | R | 0.02% (0.2) | 0% (0.0) | 0.00% |  | 0.03% (0) | 0% (0.0) | 0.00% |
| Frontal Operculum Cortex | R | 6.81% (22.8) | 0.4% (1.4) | 0.12% |  | 6.48% (22.0) | 0.46% (1.6) | 81.76% |
| Frontal Orbital Cortex | R | 1.7% (8.2) | 0.37% (1.7) | 0.19% |  | 2.34% (10.0) | 0.78% (4.2) | 47.83% |
| Frontal Pole | R | 0.84% (3.9) | 1.28% (7.7) | 0.00% |  | 0.72% (3.0) | 1.06% (6.1) | 0.44% |
| Heschl's Gyrus | R | 6.19% (19.8) | 0.33% (1.1) | 18.90% |  | 6.6% (20.0) | 0.35% (1.2) | 100.00% |
| Inferior Frontal Gyrus pars opercularis | R | 5.28% (18.7) | 0.51% (2.1) | 0.00% |  | 4.27% (16.0) | 0.4% (1.7) | 51.39% |
| Inferior Frontal Gyrus pars triangularis | R | 4.11% (15.7) | 0.37% (1.4) | 0.00% |  | 3.73% (15.0) | 0.41% (1.8) | 13.60% |
| Inferior Temporal Gyrus anterior division | R | 0.04% (0.3) | 0% (0) | 0.00% |  | 0.69% (7.0) | 0.02% (0.2) | 61.04% |
| Inferior Temporal Gyrus posterior division | R | 0.9% (5.2) | 0.16% (1.2) | 0.08% |  | 1.16% (7.0) | 0.16% (1.1) | 75.90% |
| Inferior Temporal Gyrus temporooccipital part | R | 0.47% (2.4) | 0.03% (0.1) | 0.00% |  | 0.34% (2.0) | 0.02% (0.1) | 22.94% |
| Insular Cortex | R | 6.26% (16.8) | 1.67% (4.3) | 23.95% |  | 6.23% (17.0) | 1.63% (4.3) | 98.33% |
| Intracalcarine Cortex | R | 3.74% (13.8) | 0.95% (3.9) | 3.73% |  | 2.6% (11.0) | 0.82% (3.7) | 71.67% |
| Juxtapositional Lobule Cortex | R | 1.51% (7.3) | 0.09% (0.4) | 0.00% |  | 1.42% (7.0) | 0.08% (0.4) | 42.30% |
| Lateral Occipital Cortex inferior division | R | 2.84% (9,9) | 0.81% (3.2) | 0.00% |  | 2.38% (9.0) | 0.73% (3.4) | 33.66% |
| Lateral Occipital Cortex superior division | R | 3.4% (11.5) | 1.96% (5.7) | 0.00% |  | 3.63% (12.0) | 1.92% (5.4) | 36.62% |
| Lingual Gyrus | R | 3.09% (14.6) | 0.59% (2.4) | 1.67% |  | 2.19% (12.0) | 0.52% (2.1) | 49.02% |
| Middle Frontal Gyrus | R | 2.96% (11.7) | 0.73% (2.7) | 0.00% |  | 2.47% (11.0) | 0.65% (2.9) | 4.76% |
| Middle Temporal Gyrus anterior division | R | 0.57% (4.7) | 0.05% (0.4) | 0.00% |  | 1.24% (8.0) | 0.06% (0.4) | 79.41% |
| Middle Temporal Gyrus posterior division | R | 1.15% (6.2) | 0.27% (1.6) | 0.37% |  | 1.75% (9.0) | 0.27% (1.5) | 80.81% |
| Middle Temporal Gyrus temporooccipital part | R | 1.83% (3.4) | 0.32% (1.5) | 0.20% |  | 2.05% (9.0) | 0.24% (1.0) | 80.26% |
| Occipital Fusiform Gyrus | R | 3.81% (16.1) | 0.55% (1.9) | 0.00% |  | 2.87% (13.0) | 0.47% (1.7) | 65.98% |
| Occipital Pole | R | 2.04% (7.1) | 1.02% (3.5) | 0.00% |  | 1.7% (6.0) | 0.96% (3.7) | 24.76% |
| Paracingulate Gyrus | R | 0.36% (2.1) | 0.62% (5.0) | 0.00% |  | 0.23% (2.0) | 0.34% (3.7) | 11.32% |
| Parahippocampal Gyrus anterior division | R | 0.25% (1.8) | 0.03% (0.2) | 0.99% |  | 0.3% (2.0) | 0.03% (0.2) | 40.18% |
| Parahippocampal Gyrus posterior division | R | 2.1% (12.4) | 0.06% (0.3) | 0.34% |  | 1.25% (9.0) | 0.04% (0.2) | 59.39% |
| Parietal Operculum Cortex | R | 7.94% (24.8) | 0.66% (2.4) | 6.50% |  | 8.99% (25.0) | 0.83% (2.5) | 95.07% |
| Planum Polare | R | 3.27% (12.7) | 0.22% (0.8) | 14.45% |  | 3.73% (14.0) | 0.22% (0.8) | 100.00% |
| Planum Temporale | R | 6.03% (18.7) | 0.59% (2.1) | 6.48% |  | 7.08% (21.0) | 0.55% (1.8) | 99.72% |
| Postcentral Gyrus | R | 4.22% (15.0) | 1.06% (3.5) | 0.02% |  | 4.39% (15.0) | 1.18% (3.6) | 19.97% |
| Precentral Gyrus | R | 3.73% (14.0) | 2.37% (9.3) | 0.00% |  | 3.68% (14.0) | 2.26% (8.3) | 29.25% |
| Precuneous Cortex | R | 2.44% (9,2) | 1.43% (6.1) | 0.56% |  | 2.21% (9.0) | 1.38% (6.3) | 31.70% |
| Subcallosal Cortex | R | 0% (0) | 0% (0) | 0.00% |  | 0.02% (0) | 0% (0) | 0.15% |
| Superior Frontal Gyrus | R | 0.8% (4.1) | 0.15% (0.7) | 0.00% |  | 0.83% (4.0) | 0.16% (0.7) | 12.92% |
| Superior Parietal Lobule | R | 4.38%(15.4) | 0.6% (2.0) | 0.00% |  | 4.03% (15.0) | 0.66% (2.5) | 5.65% |
| Superior Temporal Gyrus anterior division | R | 0.79% (4.1) | 0.03% (0.2) | 0.00% |  | 1.46% (8.0) | 0.04% (0.2) | 94.46% |
| Superior Temporal Gyrus posterior division | R | 2.5% (8.4) | 0.56% (2.8) | 0.66% |  | 3.28% (13.0) | 0.52% (2.5) | 81.20% |
| Supracalcarine Cortex | R | 2.9% (8.7) | 0.5% (2.1) | 0.07% |  | 2.49% (9.0) | 0.44% (2.1) | 74.61% |
| Supramarginal Gyrus anterior division | R | 6.4% (20.1) | 0.62% (2.1) | 0.01% |  | 7.00% (21.0) | 0.7% (2.2) | 32.36% |
| Supramarginal Gyrus posterior division | R | 6% (19.15) | 1.47% (5.2) | 0.31% |  | 7.04% (20.0) | 1.67% (5.4) | 30.01% |
| Temporal Fusiform Cortex anterior division | R | 0.09% (0.7) | 0% (0.0) | 0.88% |  | 0.07% (1.0) | 0% (0) | 31.84% |
| Temporal Fusiform Cortex posterior division | R | 0.29% (1.69) | 0.03% (0.2) | 7.04% |  | 0.31% (2.0) | 0.04% (0.2) | 94.23% |
| Temporal Occipital Fusiform Cortex | R | 3.16% (14.6) | 0.26% (1.1) | 1.25% |  | 2.08% (11.0) | 0.18% (0.9) | 60.82% |
| Temporal Pole | R | 0.63% (3.2) | 0.16% (0.8) | 0.02% |  | 0.84% (5.0) | 0.19% (0.8) | 56.86% |
